# Weighted-SAMGSR: combining significance analysis of microarray-gene set reduction algorithm with pathway topology-based weights to select relevant genes

**DOI:** 10.1186/s13062-016-0152-3

**Published:** 2016-09-29

**Authors:** Suyan Tian, Howard H. Chang, Chi Wang

**Affiliations:** 1 Division of Clinical Research, The First Hospital of Jilin University, 71Xinmin Street, Changchun, Jilin China 130021; 2 School of Mathematics, Jilin University, 2699 Qianjin Street, Changchun, Jilin China 130012; 3 Department of Biostatistics and Bioinformatics, Rollins School of Public Health, Emory University, 1518 Clifton Road NE, Atlanta, GA 30322 USA; 4 Department of Biostatistics, Markey Cancer Center, The University of Kentucky, 800 Rose St., Lexington, KY 40536 USA

**Keywords:** Pathway knowledge, Pathway-based feature selection, Significance analysis of microarray (SAM), Weighted gene expression profiles, Non-small cell lung cancer (NSCLC), Multiple sclerosis (MS)

## Abstract

**Background:**

It has been demonstrated that a pathway-based feature selection method that incorporates biological information within pathways during the process of feature selection usually outperforms a gene-based feature selection algorithm in terms of predictive accuracy and stability. Significance analysis of microarray-gene set reduction algorithm (SAMGSR), an extension to a gene set analysis method with further reduction of the selected pathways to their respective core subsets, can be regarded as a pathway-based feature selection method.

**Methods:**

In SAMGSR, whether a gene is selected is mainly determined by its expression difference between the phenotypes, and partially by the number of pathways to which this gene belongs. It ignores the topology information among pathways. In this study, we propose a weighted version of the SAMGSR algorithm by constructing weights based on the connectivity among genes and then combing these weights with the test statistics.

**Results:**

Using both simulated and real-world data, we evaluate the performance of the proposed SAMGSR extension and demonstrate that the weighted version outperforms its original version.﻿

**Conclusions:**

To conclude, the additional gene connectivity information does faciliatate feature selection.

**Reviewers:**

This article was reviewed by Drs. Limsoon Wong, Lev Klebanov, and, I. King Jordan.

**Electronic supplementary material:**

The online version of this article (doi:10.1186/s13062-016-0152-3) contains supplementary material, which is available to authorized users.

## Background

Many studies have demonstrated that pathway-based feature selection algorithms, which utilize biological information contained in pathways to guide which features/genes should be selected, are usually superior to traditional gene-based feature selection algorithms in terms of predictive accuracy, stability, and biological interpretation [1–10]. Consequently, pathway-based feature selection algorithms have become increasingly popular and widespread.

In contrast to a pathway analysis method, which examines the association of a whole pathway with the phenotype of interest, a pathway-based feature selection algorithm focuses on the identification of relevant individual features (e.g., genes) while considering known pathway knowledge. Pathway-based feature selection algorithms can be classified into three major categories – penalty, stepwise forward, and weighting. Their definitions and characteristics are presented in Table 1. In the penalty category, an additional penalty term accounting for the pathway structure/topology is added to the objective function for optimization. In essence, this penalty term provides some smoothness on nearby genes within a pathway, relying on the assumption that neighboring genes inside a pathway are more likely to function together or to be involved in the same biological process than those are far away. As a result, a ‘driving’ gene that has subtle changes but alters its neighbors’ expression values dramatically is more likely to be selected. One limitation of penalization methods is that they are associated with higher theoretical complexity and more computational efforts.

**Table 1:** Three categories of pathway-based feature selection algorithms. The filter and embedded methods are two typical types for the gene-based feature selection algorithms. As defined by [32], filter methods access the relevance of features by calculating some functional score while embedded methods search for the optimal subset simultaneously with the classifier construction

Category/description	Property	Pathway topology information	Examples [Ref.]
Penalty: add an extra penalty term which accounts for the pathway structure to the objective function, then optimize the resulting function to get the final gene subset	Embedded feature selection methods, carry out feature selection and coefficient estimation simultaneously, moderate to heavy computing burden	Need the pathway topology information for all genes, e.g., are they connected and the distance between them	Net-Cox [Zhang et al. 2013] netSVM [Chen et al. 2011]
Stepwise forward: order genes based on one specific statistic, and then add gene one by one until there is no gain on the pre-defined score.	Usually filter methods, the beneath concepts and theory are simple. However, they also inherits the filter methods’ drawbacks of inferior model parsimony and thus high false positive rate.	Usually ignore the pathway topology information, the decision hinges mainly on the genes’ expression values	SAM-GSR [Dinu et al, 2009] SurvNet [Li et al. 2012]
Weighting: create some kind weights according to the pathway knowledge and then combine with other feature selection methods to identify the relevant genes	With different weights, the chance of those “driving” genes with subtle change being selected increases. However, if the estimated weights subject to big biases, the resulting model might even be inferior to those without weights.	Account for the pathway topology information.	RRFE [Johannes et al. 2010] DRW [Liu et al. 2013]

The stepwise forward methods first select one gene (e.g., the most significantly differentially expressed) and add genes gradually, and then evaluate the performance of the resulting gene subset based on some statistic until no further gain upon this statistic can be obtained. The SAMGSR algorithm proposed by [11] falls within this category, and it consists of two steps. Its first step is essentially an extension of the SAM method [12] to all genes inside a pathway and the significance level of a pathway is determined using permutation tests. Then a core subset is extracted from each significant pathway identified by the first step on the basis on the magnitudes of individual genes’ SAM statistics. Based on the simulation results by us [13], SAMGSR may increase the likelihood of those genes involved in many pathways being selected. But SAMGSR only considers a gene’s pathway membership and ignores pathway topology information, it may miss those ‘driving’ genes with subtle changes because the inclusion of a gene in the reduced core subsets hinges on its expression difference between two phenotypes.

The third category is to create a pathway knowledge-based weight for each gene. For instance, the reweighted recursive feature elimination (RRFE) algorithm proposed by Johannes et al. [14] uses GeneRank [15] to calculate a rank for each gene and then weighs the coefficients in a support vector machine (SVM) model by this rank. It had been demonstrated that the resulting gene signatures have better stability and more meaningful biological interpretation [14]. Compared to the other two categories, while the weighting category is simpler, it has been underutilized. Its underutilization might be due to the estimated weights being subject to errors and biases where the impact on the resulting significant may be substantial.

In this study, we propose a hybrid method that combines SAMGSR with a pathway topology-based weight to carry out feature selection. As a combination of the weighting method and stepwise forward method, the objective is to address some disadvantages associated with SAMGSR and the weighting method while utilizing their strengths. The proposed method is referred to as weighted-SAMGSR herein. Applying it to both the simulated data and real-world application, we evaluate if weights reflecting gene connectivity information are valuable for feature selection.

## Methods

### Experimental data

We considered two sets of microarray data and one RNA-Seq dataset in this study. One set of microarray data is for a multiple sclerosis (MS) application; the other set and the RNA-Seq data are for a non-small cell lung cancer (NSCLC) application.

### MS data

The MS application consisted of two microarray experiments. The first one included chips from the experiment E-MTAB-69 stored in the ArrayExpress [16] repository (http://www.ebi.ac.uk/arrayexpress). All chips were hybridized on Affymetrix HGU133 Plus 2.0 chips. In this study, there were 26 patients with relapsing-remitting multiple sclerosis (RRMS) and 18 controls with neurological disorders of a non-inflammatory nature. The second dataset was provided by the sbv IMPROVER challenge in the year of 2012 [17], which is accessible to the participants on the project website (http://www.sbvimprover.com). It was hybridized on Affymetrix HGU133 Plus 2.0 platform, and there were 28 patients with RRMS and 32 normal controls.

### NSCLC data

In the non-small cell lung cancer (NSCLC) application, we considered two cases: the two-class case and the multiple-class case. For the two-class case, the RNA-Seq data for those patients at early histology stages (stages I and II) were downloaded from The Cancer Genome Atlas (https://tcga-data.nci.nih.gov/tcga/) and served as the training set. One microarray NSCLC data in the Gene Expression Omnibus (GEO) repository (accession No. GSE43580) was used to validate the results.

For the multiple-class case, several microarray data were used as the training set. They included the data deposited under accession numbers of GSE10245, GSE18842, and GSE2109 in the GEO repository. All these 3 experiments were hybridized on the Affymetrix HGU133Plus 2.0 chips. Then GSE43580 was used again to evaluate the performance of both SAMGSR algorithms.

### Gene sets

Gene sets were downloaded from the **Molecular Signatures Database** (MSigDB) [18]. In this study, we only considered the c5 category. This category includes gene sets annotated by Gene Ontology (GO) terms. The current version (version 4.0) of MSigDB c5 category included 1454 gene sets.

### Pre-processing procedures

Raw data of E-MTAB-69 were downloaded from the ArrayExpress repository, and expression values were obtained using the fRMA algorithm [19] and normalization across samples was carried out using quantile normalization. The resulting expression values were on the log_2_ scale. When there were multiple probe sets representing the same gene, the one with the largest fold change was chosen. Raw data of the second set were downloaded from the sbv challenge website, and were separately pre-processed in the same way.

For the NSCLC RNA-seq data, Counts-per-million (CPM) values were calculated and log_2_ transformed by Voom function [20] in the R limma package [21]. Raw data (CEL files) of all NSCLC microarray data sets were downloaded from the GEO repository, and expression values were obtained using the fRMA algorithm [19]. Since the training set included data from different microarray experiments, the COMBAT algorithm (http://www.bu.edu/jlab/wp-assets/ComBat/Abstract.html) was used to eliminate batch effects. In both two-class and multiple-class cases, the training sets and the test sets were pre-processed separately.

### Statistical methods

#### SAMGSR

SAMGSR extends a pathway analysis method called significance analysis of microarray-gene set (SAMGS) [22] to identify the ‘core’ subset for each significant gene set. In SAMGS, the following functional score is defined,

$$ SAMG{S}_j\kern0.5em =\kern0.5em {\displaystyle \sum_{i=1}^{\left|j\right|}{d}_i^2\kern0.5em ,{d}_i\kern0.5em =\kern0.5em \left(\overline{x_d}(i)-\overline{x_c}(i)\right)/\left(s(i)+{s}_0\right)} $$

where d_i_ is the SAM statistic [12] and calculated for each gene involving in gene set *j,*${\overline{x}}_d(i)$ and ${\overline{x}}_c(i)$ are the sample averages of gene *i* for the diseased and control group, respectively. Parameter s(i) is a pooled standard deviation that is estimated by pooling all samples together, while s_0_ is a small positive constant used to offset the small variability in microarray expression measurements, and |j| represents the number of genes within gene set *j*. A gene set’s significance is estimated using a permutation test by perturbing phenotype-labels.

**Fig. 1:**
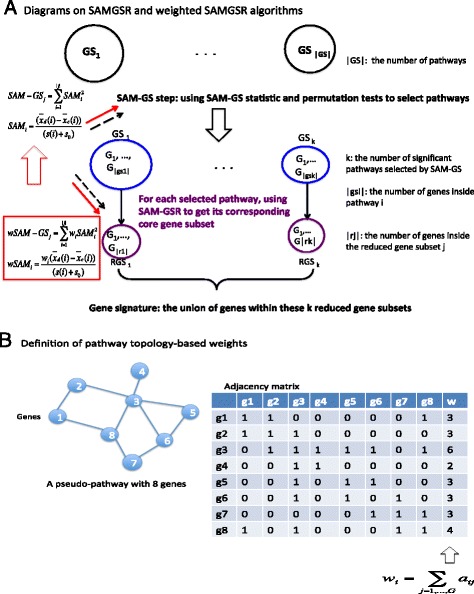
Diagrams to elucidate both SAMGSR and weighted-SAMGSR algorithms

For each significant gene set identified by SAMGS, the genes inside the set are ordered decreasingly based on the magnitude of d_i_. The additional reduction step of SAMGSR gradually partitions the entire set into two subsets: the reduced subset *R*_*k*_ including the first k genes and the residual one ${\overline{R}}_k$ including the remaining genes for k = 1,…, |j|. At each partition, the significance level of ${\overline{R}}_k$ was evaluated using the SAM-GS *p*-value of ${\overline{R}}_k$. The iteration stops when this *p*-value is larger than a pre-determined threshold c_k_ for the first time. Figure 1 provides a graphical illustration of the SAMGSR algorithm.

#### Weighted-SAMGSR

**Fig. 2:**
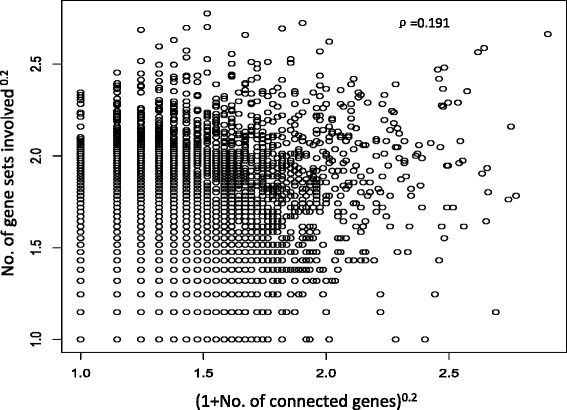
Scatterplot to show the correlation between the number of gene sets one gene is involved and its connectivity. ρ is the estimated Spearman correlation coefficient between the number of gene sets involved and (1 + the number of connected genes)

SAMGSR only assumes the genes within a specific pathway would function together to impact a biological process. In SAMGSR, the larger number of gene sets in which a specific gene is, the larger this gene’s probability to be selected may be. From the scatterplot (Fig. 2), we found a moderate positive correlation (Spearman’s correlation coefficient = 0.191) between the number of gene sets a gene is involved in and its connectivity level with other genes, indicating the interactions among genes provide additional information rather than that provided by the number of gene sets being contained.

To tackle the ignorance of SAMGSR on gene connectivity information, we propose to combine a weight constructed on the basis of connectivity information with the SAMGS statistic. Specifically for G genes under consideration, a G × G adjacency matrix is defined. Its *ij* component a_ij_ equals to 1 if genes *i* and *j* are connected, 0 otherwise. Because here we only consider an undirected pathway connectivity diagram, this adjacency matrix is symmetric. Then the connectivity weight for gene *i* is defined as,

$$ {w}_i\kern0.5em =\kern0.5em {\displaystyle \sum_{j=1,\dots, G}{a}_{ij}\kern0.5em ,\kern0.5em {a}_{ii}\kern0.5em =\kern0.5em 1} $$

by setting α_ii_ = 1, a gene is set to be self-connected and avoids zero weights for those isolated genes.

In our proposed procedure, we include each gene’s weight in its SAM statistic to obtain so-called weighted SAM and weighted SAMGS statistics and then replace SAM/SAMGS with their weighted counterparts to execute pathway selection followed by individual gene selection. The proposed method is referred to as weighted-SAMGSR herein. In Fig. 1, the definition of weighted SAMGS statistics and where they replace SAMGS statistics are presented. Within each specific pathway, SAMGSR ranks genes based on their SAM statistics. In contrast, the weighted-SAMGSR algorithm assigns genes with high connectivity more weights. This is motivated to better detect the ‘driving’ genes that are highly connected to other genes but have subtle expression differences.

In both SAMGSR and weighted-SAMGSR, the cut-off value for c_k_ is regarded as a tuning parameter that determines the sparseness of the final models. Its optimal values are determined via k-fold cross-validations (CVs) by randomly dividing the whole training dataset into k roughly equal-sized folds. We apply either SAMGSR or weighted-SAMGSR to k-1 of these folds and verify their performance on the held-out fold. This step is repeated for each of the k folds as the held-out fold, and then the error rate is calculated. We then take the optimal cut-off value and apply SAMGSR or weighted-SAMGSR to the whole training dataset to select genes in the final models, whose performances are evaluated using independent test sets. Of note, since for the SAMGSR methods the classifiers are not automatically produced along with the process of feature selection, we fit support vector machine (SVM) models to estimate the corresponding coefficients of the selected genes.

The connectivity information was retrieved from two databases: 1) the Human Protein Reference Database (HPRD) where the protein-to-protein interaction (PPI) information was downloaded from the HPRD webpage (www.hprd.org), and then the adjacency matrix among genes was calculated using the R software; and 2) the STRING software (www.string-db.org), using both the connectivity among genes and the scores representing the confidence level on those connections.

### Statistical metrics

As in previous study [23], we use four metrics - Belief Confusion Metric (BCM), Area Under the Precision-Recall Curve (AUPR), Generalized Brier Score (GBS), and misclassified error rate to evaluate the performance of a resulting gene signature. Specifically, the misclassified error rate is simply calculated as (false positives + false negatives/total samples. The cut-off for the confidence values is set at 0.5, i.e., when a confidence value >0.5 for a given class then this sample is classified into that class. For GBS, we used the equation given by Yeung et al [24], and then further scaled it to the interval of 0 to 1 by dividing the sample size n,

$$ GBS=\kern0.5em \frac{1}{2n}{\displaystyle {\sum}_{i=1}^n{\displaystyle {\sum}_{k=1}^K{\left({Y}_{ik}-{p}_{ik}\right)}^2}} $$

where Y_ik_ (1 if subject i belongs to class k, and 0 otherwise) is an indicator function for subject i (*i* = 1,…,n) in class k (*k* = 1,…,K), and p_ik_ denotes the predicted probability for subject i in class k.

BCM and AUPR are two metrics used in the sbv Improver challenge. Using BCM and AUPR may represent a fair comparison between the weighted-SAMGSR algorithm and the top-performed teams in this challenge. BCM is defined as,

$$ BCM\kern0.5em =\kern0.5em 1-\frac{1}{K}\left[\left(1-\frac{v_1}{N_1}\right)\kern0.5em +\cdot \cdot \cdot +\left(1-\frac{v_k}{N_k}\right)\right] $$

which captures the average confidence that a sample belongs to class k when it indeed belongs to class k. Then AUPR computed as the average over all classes k of the AUPR_k_ for each class. The precision is defined as true positives/(true positives + false positives) while the recall as true positives/(true positives + false negatives). The AUPR_k_ was computed by sorting the list of subjects in class k according to their confidences/probabilities. AUPR captures the ability of correctly ranking the samples known to belong in a given class.

Besides these predictive performance statistics, we additionally include the Rand index to evaluate the stability or robustness of the resulting signatures. With k runs of an algorithm, Rand index is defined as

$$ Rand=\frac{2}{k\left(k-1\right)}{\displaystyle \sum_{i=1}^{k-1}{\displaystyle \sum_{j=i+1}^k\frac{\cap \left(\left|g{s}_i\right|,\left|g{s}_j\right|\right)}{\cup \left(\left|g{s}_i\right|,\left|g{s}_j\right|\right)}}} $$

where ∩ represents the size of intersection between two gene lists and ∪ represents the size of union between two gene lists gs_i_ and gs_j_ obtained from the i^th^ and j^th^ runs. Rand index can also be defined at the level of pathways by replacing the gene lists with the pathway lists.

### Statistical language and packages

All statistical analysis was carried out in the R language version 3.1 (www.r-project.org).

## Results

### Simulated data

Two simulations were used to characterize the weighted-SAMGSR algorithm and to make comparisons with the SAMGSR algorithm. Here, we randomly chose 5 gene sets in the MSigDB c5 category. There are approximately 1000 genes inside these 5 gene sets. In the first simulation, we simulated the gene expression profile as independent random variables with a standard normal distribution and the sample size was 60. Then we simulated another set of normally distributed random variables and used it as the test set. In the second simulation, the observed expression values of the integrated NSCLC microarray data were used to train the final model. The expression values were further normalized to have means of zeros and standard deviations of ones. The standardized expression values of GSE43580 were used to test the final model and evaluate its performance.

We chose two genes—*HDAC1* and *GNAS* as the relevant genes and simulated the case and control groups using the following logit function,

$$ logi{t}_{2vs1}\kern0.5em =\kern0.5em 0.37{X}_{HDAC1}-0.86{X}_{GNAS} $$

because *HDAC1* has the higher connectivity, its coefficient was set as being smaller than that of *GNAS*. The simulation results are presented in Table 2.

**Table 2:** Simulation results

	Training set		Test set			
Method (Size^a^)	HDAC1 (%)	GNAS (%)	Error (%)	GBS	BMC	AUPR
A. Simulated from 60 independent normal-distributed random variables						
SAMGSR (3.8)	19	100	16.5	0.118	0.733	0.921
W-SAMGSR (6.23)	65	100	13.2	0.101	0.755	0.948
B. Simulated based on the NSCLC microarray data						
SAMGSR (3.94)	0	100	44.5	0.256	0.517	0.550
W-SAMGSR (6.28)	77	100	40.5	0.241	0.534	0.621

*Note*: W-SAMGSR stands for weighted-SAMGSR

^a^stands for average the number of genes selected by either SAMGSR or W-SAMGSR over 100 replicates

Overall, the weighted-SAMGSR algorithm outperforms the SAMGSR algorithm. Specifically, the weighted-SAMGSR algorithm has a substantially higher probability to identify HDAC1 whose signal is about 1.5 times weaker than GNAS and has better performance statistics in these two scenarios. Therefore, constructing the weights based on genes’ topology information and combining those weights with the SAMGS statistics improve upon the performance of SAMGSR in terms of correctly selecting true relevant genes and predictive ability.

### Real world data

In this study, we use three applications to evaluate if weighted-SAMGSR is superior to SAMGSR by accounting for the additional connectivity information among genes.

Both the MS and NSCLC multi-class applications are two sub-tasks of the sbv Improver challenge, 2012 [17]. Considered there are 54 teams participated in the challenge and participants used various feature selection and classification methods, the best performing teams in this challenge may indicate the upper limit of a gene signature/classifier for those data/applications, and the methods those top-ranked teams used may be considered as the most suitable ones for those data/applications. A comparison of the weighted-SAMGSR algorithm with those best performing teams is very meaningful. Therefore, we listed the predictive statistics of those top-ranked teams and made a comparison between them and the weighted-SAMGSR algorithm. Furthermore, two pathway-based feature selection algorithms — RRFE [14] and generalized elastic net (gelnet) [25] plus two widely-used gene-based methods — LASSO [26] and penalized support vector machine (SVM) [27] were considered, and then compared with both SAMGSR methods.

### Two-class cases

#### MS data

MS is the most prevalent demyelinating disease and the leading cause of neurological disability in young adults [28]. Here, we analyzed a set of MS real-world data to explore the discriminative capacity of expression profiles to separate MS patients from controls, and to characterize the proposed weighted-SAMGSR method. Here, the connectivity information was retrieved from the HPRD database.

The results are presented in Tables 3 and 4. In Table 3, we observe the selected pathways by SAMGSR and weighted-SAMGSR with high frequencies differ considerably. On the level of individual genes, there are 6 overlapped genes. According to the genecards (www.genecards.org) database, two genes – *POLD1* and *MRE11A* among these 6 genes are directly related with MS. In terms of stability, the weighted-SAMGSR algorithm shows a slight increment over the SAMGSR algorithm, i.e., 14.03 % versus 12.83 % at the gene level and 15.76 % versus 14.04 % at the gene set level.

**Table 3:** Selected pathways and genes on MS data

	Pathways with high frequency (frequency %)	Genes (frequency %)	
SAMGSR	DNA Directed DNA Polymease Activity (100 %)	**POLD4 ** (100 %)	**POLD1 ** (80 %)
	DNA Polymease Activity (90 %)	PHB (80 %)	**GPAA1 ** (70 %)
	COVALENT_CHROMATIN_MODIFICATION (70 %)	**PIGT** (70 %)	DPM3 (70 %)
	HISTONE_MODIFICATION (70 %)	**MRE11A** (60 %)	**PI4KB** (60 %)
	Stability = 14.04 %	Stability = 12.83 %	
Weighted-SAMGSR	DNA_RECOMBINATION (70 %) LIPOPROTEIN_BIOSYNTHETIC_PROCESS (70 %)	**MRE11A** (90 %)	PTPRC (80 %)
	NEGATIVE_REGULATION_OF_IMMUNE_SYSTEM_PROCESS (70 %)	BRCA1 (70 %)	ATM (70 %)
	PROTEIN_AMINO_ACID_LIPIDATION (70 %)	CHAF1A (70 %)	**PIGT** (70 %)
	DEPHOSPHORYLATION (60 %) INOSITOL_OR_PHOSPHATIDYLINOSITOL_KINASE_ACTIVITY (60 %)	**GPAA1** (70 %)	**PI4KB** (70 %)
	LIPOPROTEIN_METABOLIC_PROCESS (60 %) PROTEIN_C_TERMINUS_BINDING (60 %)	PEX16 (60 %)	**POLD1** (60 %)
		**POLD4** (60 %)	PPP1CA (60 %)
	Stability = 15.76 %	Stability = 14.03 %	

*Note*: Gene symbols in bold are those overlapped genes by SAMGSR and weighted-SAMGSR; gene symbols underlined are directly related to MS according to the genecards database

**Table 4:** Performance statistics of selected genes on MS data

	Training set (10-fold CV results)				Test set			
A. Performance comparison								
Method (n)	Error (%)	GBS	BCM	AUPR	Error (%)	GBS	BCM	AUPR
SAMGSR (52)	34.09	0.244	0.570	0.645	46.67	0.465	0.501	0.725
W-SAMGSR (25)	31.82	0.191	0.611	0.771	43.33	0.341	0.564	0.860
LASSO (30)	34.09	0.275	0.632	0.672	46.67	0.377	0.499	0.747
Penalized SVM(11)	47.73	0.406	0.534	0.630	45	0.569	0.431	0.555
gelnet (169)	34.09	0.251	0.528	0.589	46.67	0.246	0.547	0.746
RRFE (198)	43.18	0.263	0.547	0.619	46.67	0.300	0.523	0.693
B. Performance of the top 3 teams in sbv MS sub-challenge (among 54 teams)								
Study (size)	Training data used/Method used				Error (%)	GBS	BCM	AUPR
Lauria’s (*n* > 100)	E-MTAB-69/Mann-Whitney test, then use top α % of the selected genes and Cytoscape to get the clusters on the test set				--	--	0.884	0.874
Tarca’s (*n* = 2)	GSE21942 (on Human Gene 1.0 ST)/LDA				--	--	0.629	0.819
Zhao’s (*n* = 58)	7 other data and E-MTAB-69/Elastic net				30	--	0.576	0.820

*Note*: *W-SAMGSR* weighted-SAMGSR, *LDA* linear discrimination analysis, *gelnet* generalized elastic net by [25], *RRFE* reweighted recursive feature elimination by [14]

--: not available. Lauria’s Tarca’s and Zhao’s studies [38, 39, 44] are the 3 best studies in the sbv MS sub-challenge

As shown in Table 4, the performance of SAMGSR is substantially inferior to that of the top 3 teams in the sbv challenge. After taking the extra information of gene connectivity into consideration, the performance of the weighted-SAMGSR algorithm becomes comparable to the third team in this sub-challenge. In summary, weighted-SAMGSR outperforms SAMGSR in terms of predictive performance and stability.

#### NSCLC data

NSCLC accounts for approximately 85 % of the lung cancer cases [29]. Lung cancer is a multistage progression process resulted from genetic sequences mutations, and thus it is postulated that genes associated with NSCLC patients at histology stage I and with those at stage II might differ potentially. In this application, we explored the discriminative capacity of expression profiles to separate NSCLC stage I patients from stage II patients by training both SAMGSR algorithms on a NSCLC RNA-Seq data set.

From Table 5, we found there is no overlap between the selected pathways by SAMGSR and weighted-SAMGSR at high frequencies. On the level of individual genes, only *CFTR* and *TGFB2* are identified by both algorithms, indicating these 2 genes not only have large expression differences between stage I and II but also high connectivity with other genes. According to the genecards database, only *CFTR* is directly related to NSCLC. Compared to the SAMGSR algorithm, the stability of the weighted-SAMGSR algorithm improves substantially: the Rand index over 10 CV runs increases from 18.28 to 42.15 % on the gene set level, and increases from 24.48 to 32.38 % on the gene level.

**Table 5:** Selected pathways and genes on NSCLC RNA-seq data (stage segmentation)

	Pathways with high frequency (frequency %)	Genes (frequency %)
SAMGSR	DNA_FRAGMENTATION_DURING_APOPTOSIS (70 %) SODIUM_CHANNEL_ACTIVITY (70 %)	**TGFB2** (80 %) SHROOM2 (80 %) CECR2 (70 %) SCN4B (70 %) **CFTR ** (70 %)
	Stability = 18.28 %	Stability = 24.48 %
Weighted-SAMGSR	ANION_CHANNEL_ACTIVITY (100 %) CHLORIDE_CHANNEL_ACTIVITY (100 %) ANION_TRANSMEMBRANE_TRANSPORTER_ACTIVITY (90 %) AXON (90 %) APICAL_PART_OF_CELL (90 %) NEURON_PROJECTION (90 %) ANION_TRANSPORT (80 %) REGULATION_OF_MAPKKK_CASCADE (80 %) GROWTH_CONE (80 %) REGULATION_OF_HEART_CONTRACTION (80 %) REGULATION_OF_MUSCLE_CONTRACTION (80 %) SITE_OF_POLARIZED_GROWTH (70 %) PROTEIN_FOLDING (70 %)	**CFTR** (100 %) **TGFB2** (80 %) MAPT (80 %) MAPK8IP3 (70 %) TPM1 (70 %)
	Stability = 42.75 %	Stability = 32.38 %

*Note*: Gene symbols in bold are those overlapped genes by SAMGSR and weighted-SAMGSR; gene symbols underlined are directly related to NSCLC according to the genecards database

Consistent with the results from the sbv Lung cancer (LC) challenge [30], two NSCLC early stages cannot be separated distinctly from each other. Nevertheless, weighted-SAMGSR outperforms SAMGSR with respect to all four predictive statistics, as shown in Table 6.

**Table 6:** Performance statistics of selected genes on NSCLC RNA-seq data (stage segmentation)

	Training set (10-fold CV results)				Test set			
Method (n)	Error (%)	GBS	BCM	AUPR	Error (%)	GBS	BCM	AUPR
SAMGSR (9)	35.2	0.242	0.539	0. 575	50	0.279	0.507	0.531
W-SAMGSR (8)	32.8	0.231	0.556	0.584	49.3	0.276	0.513	0.580
LASSO (30)	36	0.219	0.558	0.610	50	0.453	0.500	0.509
Penalized SVM (34)	36.8	0.255	0.562	0.603	50	0.329	0.501	0.518
gelnet (252)	36.8	0.231	0.517	0.547	50	0.465	0.499	0.475
RRFE (93)	35.2	0.185	0.545	0.578	50	0.471	0.500	0.506

*Note*: *W-SAMGSR* weighted-SAMGSR, *gelnet* generalized elastic net, *RRFE* reweighted recursive feature elimination

### Multiple-class case

Both the SAMGSR algorithm and the weighted-SAMGSR algorithm can be adopted directly to deal with the multiple classes (>2 groups). Here, we used a set of NSCLC microarray data to showcase this. In this application, the patients were categorized into four classes according to their respective histology subtypes and clinical stages, i.e., adenocarcinoma at stage I (AC-I), adenocarcinoma at stage II (AC-II), squamous cell carcinoma at stage I (SCC-I), and squamous cell carcinoma at stage II (SCC-II). To classify on these four groups, we applied both SAMGSR algorithms twice — one for the subtype segmentation and the other for the stage segmentation. Then the final posterior probabilities are P(AC-I) = P(AC) × P(stage I), P(AC-II) = P(AC) × P(stage II), P(SCC-I) = P(SCC) × P(stage I), and P(SCC-II) = P(SCC) × P(stage II), respectively. The results are given in Table 7.

**Table 7:** Performance statistics of selected genes on NSCLC data (multiple-class case)

	Training set (5-fold CV results)				Test set			
A. Performance comparison								
Method (n)	Error (%)	GBS	BCM	AUPR	Error (%)	GBS	BCM	AUPR
SAMGSR (30)^a^	40.7	0.279	0.377	0.462	51.3	0.348	0.407	0.486
W-SAMGSR (27)^a^	37.2	0.276	0.378	0.453	51.3	0.345	0.405	0.492
LASSO (95)	38.6	0.281	0.458	0.483	52.7	0.395	0.456	0.485
pSVM (>100)	42.8	0.370	0.344	0.428	53.3	0.433	0.385	0.397
gelnet (>400)	36.6	0.284	0.346	0.416	54.7	0.343	0.377	0.489
RRFE (>200)	36.6	0.272	0.395	0.448	54	0.336	0.410	0.468
B. Performance of the top 3 teams in sbv NSCLC sub-challenge (among 54 teams)								
Study (size)	Training data used/Method used				Error (%)	GBS	BCM	AUPR
Ben-Hamo’s (23)	GSE10245, GSE18842, GSE31799/PAM				49.3	--	0.48	0.46
Tarca’s (25)	GSE10245, GSE18842, GSE2109/moderated t-tests + LDA				--	--	0.459	0.454
Tian’s (66)	GSE10245, GSE18842, GSE2109/TGDR in hierarchical way				53.3	0.374	0.440	0.471

*Note*: *W-SAMGSR* weighted-SAMGSR, *pSVM* penalized support vector machine (SCAD penalty term), *gelnet* generalized elastic net, *RRFE* reweighted recursive feature elimination, *LDA* linear discriminant analysis, *PAM* partitioning around medoid, *TGDR* threshold gradient descent regularization

^a^The sizes of final model for the stage segmentation because the results for the subtype segmentation for both algorithms are identical (but the final size > 300). Ben-Hamo’s study [31], Tarca’s study [44] and Tian’s study [45] are the 3 best studies in the sbv LC sub-challenge

Consistent with the results from the sbv Lung cancer (LC) challenge [30], the segmentation between stages is not achievable whereas the segmentation between subtypes is good. Nevertheless, both SAMGSR algorithms identify more than 300 genes for the subtype segmentation while other studies had obtained similar performance using just one [31] or several genes [23]. We attribute this to the facts that: 1) SAMGSR is a filter method [32] that screens the genes one by one and thus tends to introduce all highly correlated genes to the true relevant ones into the final model; and 2) the sample size of this application is relatively large and the SAM statistic, and consequently SAMGS statistic, are very sensitive to the sample size. A statistically insignificant difference between two phenotypes with a small sample size would be regarded as significance when the sample size is considerably large. Also, the expression difference between AC and SCC is indeed very distinct, compared to that between stage I and stage II.

By accounting for the connectivity among genes, weighted-SAMGSR outperforms SAMGSR in these three real-world applications, which is consistent with the results from the simulated data. Nevertheless, it is observed that such superiority differs in these applications — being substantial in the MS application whereas marginal in the NSCLC application. This may be attributable to that many cancers are under intensive investigation and may be better curated in the major pathway databases. Therefore, the genes inside one specific pathway might be more likely to function together for the cancer cases, making the underlying assumption of the SAMGSR algorithm more reasonable.

It is also observed that the weighted SAMGSR algorithm performs comparable to or better than other feature selection algorithms under consideration, i.e., LASSO, penalized SVM, gelnet, and RRFE with respect to the predictive ability. Regarding to the stability on the gene level, the weighted SAMGSR algorithm is ranked second and only outperformed by gelnet that has a Rand index of 22.52 and 33.66 % in the MS application and the NSCLC application, respectively. Since both LASSO and gelnet implement the estimation process using the cyclic coordinated descent method [33], they require the least computing time. Penalized SVM and RRFE are the most computationally intensive, while both SAMGSR algorithms fall in the middle.

In addition, the PPI information in the STRING database [34] were used to construct the adjacent matrixes and to illustrate the utility of the weighted-SAMGSR algorithm, while different databases may contain different gene connectivity information. Table 8 presents the performance statistics for the weighted-SAMGSR algorithm using both the connectivity information and the confidence values for those interactions. The conclusion that the weighted-SAMGSR algorithm is superior to the SAMGSR algorithm remains persistently true for all three applications. Moreover, the performance of weighted-SAMGSR using the confidence values tends to be better than that using the dichotomized values. Further investigations should consider the choice of databases to retrieve the gene connectivity information and which values for the weights for one specific application.

**Table 8:** Performance statistics on the test set for the weighted-SAMGSR algorithm (PPI information retrieved from the STRING database)

	No.	Error (%)	GBS	BMC	AUPR	Rand (gene)	Rand (GS)
MS (b)	22	43.3	0.279	0.581	0.847	15.3 %	27.1 %
MS (c)	20	28.3	0.179	0.613	0.828	15.5 %	25.4 %
Stage for LC (b)	32	45.3	0.318	0.520	0.552	36.3 %	40.1 %
Stage for LC (c)	26	45.3	0.274	0.525	0.566	35.8 %	40.4 %
MC for LC (b)	22^a^	47.3	0.337	0.411	0.510	--	--
MC for LC (c)	31^a^	51.3	0.334	0.410	0.512	--	--

Note: (b): using the binary values indicating if two genes are connected or not; (c): using the confidence scores for the gene connectivity. MS: the multiple sclerosis application; Stage for LC: the NSCLC stage application trained on the RNA-Seq data; MC for LC: the NSCLC multiple-class application. Rand (gene): the rand index at the gene level, across the gene lists obtained from 10-fold cross-validation data; Rand (GS): the rand index at the gene set level

^a^is the number of selected genes for the stage segmentation, the number of selected genes for the subtype segmentation > 300

## Conclusions

Although SAMGSR is a pathway-based feature selection algorithm in nature, it treats all genes inside one pathway equally and assumes the genes in one specific pathway co-function together to regulate biological processes. To tackle its major drawback of discarding the gene topology knowledge, we propose a weighted extension to SAMGSR by creating weights based on the connectivity among genes and combining those weights with the SAMGS statistics. Using simulations and multiple real-world applications, we demonstrated that this weighted version of SAMGSR outperforms the original SAMGSR algorithm. In addition, the weight construction is very straightforward and has added little computational burden to the algorithm. Therefore, the weighted-SAMGSR algorithm is preferred over the SAMGSR algorithm.

Currently, pathway-based feature selection algorithms have become a topic of increasing interest in the field bioinformatics. Incorporating additional meaningful information does facilitate feature selection [25]. However, the pathway knowledge is far from completeness and thus is subject to changes and errors, which limits the use of those pathway-based methods in practice. To address this, we recommend analyzing the real-world data with both gene-based methods and pathway-based methods such as the weighted-SAMGSR algorithm and to explore if the pathway knowledge is informative for the specific data.

## Reviewers’ reports Round 1

### Reviewer’s report 1: Dr. Limsoon Wong, National University of Singapore, Singapore

#### Reviewer summary

1/This paper is badly organized and badly written, and the abstract is written in a way that lacks appeal. This makes the paper unattractive to readers. 2/The performance evaluation is limited and unconvincing. In particular, the cross-dataset error rate was ~40 %, which is unusable; and the stability of feature selection was ~14 %, which is essentially no stability. Furthermore, it was not compared to convincing competing methods. This makes the method unattractive for practical use. 3/There is not much discussion and analysis of results. This makes it difficult for readers to gain much useful insights. 4/The review of existing methods seems to have omitted a major category (which includes famous geneset-based methods like GSEA), and there is little effort to articulate the novelty of the proposed method against the very extensive collection of past works.

#### Reviewer recommendations to authors

1/This paper is badly organized and written. The followings are some major presentation issues (and there are many other presentation issues).

- The proposed weighted-SAMGSR method is not described clearly and is not described under the method section. Its description got mixed up in the results section. Its novelty is not articulated and, whatever the novelty is, its contribution to improving performance is not very well explained and evaluated in depth.

Author’s response: *We have moved the description on the weighted-SAMGSR method to the****Methods****section, and added additional analyses to explore the proposed method more comprehensively.*

- The existing method SAMGSR that it claims to improved on, as described in the method section, is different from that described in the intro and results section. In particular, the one in the methods section says nothing about “SAMGSR assumes that the number of gene sets within which a specific gene is contained is highly correlated with the pathway connectivity” and does not say how it ranks genes across significant gene sets (it just ranks gene sets).

Author’s response: *Based on our experience of using SAMGSR, the probability to be selected is positively correlated to the number of gene sets this particular gene is involved. To clarify this, we have rephrased the corresponding sentences.*

In the reduction step of SAMGSR, the genes within a specific selected gene set were ordered according to its SAM statistic (or more precisely, the *p*-value of its SAM statistic). Starting from the gene with the most significant *p*-value, the whole gene set was divided into two subset: the reduced subset and its complement, then the *p*-value of SAMGS statistic for the complement set is compared with a cutoff value c_k_. If this *p*-value is less than c_k_, the next gene is added to the reduced subset and deleted from the complement set. This step iterates until this *p*-value is larger than c_k_ for the first time. The selected genes by SAMGSR are the union of genes inside those reduced sets.

- The stability is evaluated by Rand index for k runs. It is not clear what k runs is. For each disease you have a pair of datasets. I suppose *k* = 2? Also, other evaluation metrics are named but not defined in the manuscript; not self contained.

Author’s response: *Sorry for not stating this explicitly in the manuscript. k refers to the number of folds in the cross-validation. For the MS and NSCLC two-case applications, we conducted a 10-fold cross-validation and thus k = 10.*

Regarding other metrics used, we have added their definitions in the **Methods** section.

2/The performance evaluation is limited and unconvincing.

- More datasets should be tried.

Author’s response: *We have applied the weighted-SAMGSR algorithm to more datasets. Specifically, a non small-cell lung cancer (NSCLC) RNA-Seq data set was tried, as suggested by Reviewer 3.*

- The error rate and stability reported are unimpressive. Cross-dataset error rate was ~40 %, not much better than random and hence unusable. Stability was ~14 %, also not much better than random, and would be far below those of e.g. PFSNet [35], ESSNET [36], or GAT [37].

Author’s response: *Ideally, a Rand Index, a BCM, and an AUPR being closer to 1’s while a GBS and an error rate being closer to 0 represent a better segmentation between classes. In practice, however, the best scores on these metrics depend on the applications. Thus we believe that the relative values of those metrics play a more important role when comparing across methods. In terms of those performance metrics, the weighted-SAMGSR algorithm has been demonstrated to outperform the SAMGSR algorithm. (Of note, the error rate is a threshold-dependent metric. Here, we used 0.5 as the cutoff, which might not be the optimal one.)*

Specifically, for the stability (i.e., the Rand index), we took the average over 45 pairs (for the 10 gene lists obtained from 10-fold CVs). Considered the sample size of the training set is just moderate, it is non-surprising that the values of Rand index are small. Rand index depends on applications, too. For example, the pathway-level agreement of PFSNet is 100 % for the leukemia application but is 56 % for the ALL application (as shown in ref. [35]). The weighted-SAMGSR algorithm is superior to the SAMGSR algorithm in terms of stability.

- The performance is compared against a few past methods. But it is not clear that these methods are the best or more well known ones that use gene set or pathway information.

Author’s response: *The reason we chose the MS and NSCLC multi-class applications to illustrate the proposed method is because they are two sub-tasks of the sbv Improver challenge, 2012. Considered there are 54 teams participated in the sbv challenge and participants used various feature selection and classification methods, we think the best performing teams in this challenge may indicate the upper limit (the best performance) of a gene signature/classifier for those data/applications, and the methods those top-ranked teams used may be considered as the most suitable ones for those data/applications. Therefore, a comparison of the weighted-SAMGSR with those best performing teams may be more valuable. Notably, among the best performing teams, several of them account for the pathway information, e.g.,* [38] *and* [39]*.*

3/Past works cited on gene set-based methods strangely omits famous ones like GSEA, Irizarry et al., etc. In fact, the 3 categories into which the authors put past gene set-based methods into don’t fit these famous methods.

Author’s response: *We defined a pathway-based feature selection method differently from a gene set analysis method, which explores the association between a whole pathway and the phenotype of interest. In contrast, a pathway-based feature selection algorithm incorporates pathway knowledge to guide the selection of****individual genes****that are associated with the phenotype. Based on these definitions, GSEA and other well-known methods like Pathifier* [40] *and SAMGS* [22] *are classified as a gene set analysis method, which concerns about the identification of relevant pathways. Therefore, they were not included in the****Background****section.*

We are sorry for not emphasizing this essential difference between a gene set analysis method and a pathway-based feature selection method. To address this, we have clarified on this specifically and explicitly in the **Background** section.

4/The background emphasizes stability of feature selection. However, the evaluation of stability is quite limited and not emphasized/presented very well.

Author’s response: *Sorry for this inconsistency. We have added more evaluation on the stability using the NSCLC application (stage I versus II segmentation using RNA-seq data) and discussed more about the corresponding results.*

5/There is not much discussion and analysis of results. Without an in-depth serious discussion and analysis of results, it is difficult for the reader to gain much insight.

Author’s response: *We have added more discussion on the results.*

#### Minor issues

The English is very poor. Too many mistakes to list here.

Author’s response: *We have edited the English exclusively.*

### Reviewer’s report 2: Dr. Lev Klebanov, Charles University, Czech Republic

#### Reviewer summary

Authors did not mentioned some problems connected to normalization procedure and to multidimensional character of pathways. They have to add corresponding information into the paper.

#### Reviewer recommendations to authors

The authors describe their aims as follows: “In this study, we propose a hybrid method that combines SAMGSR with a pathway topology-based weight to carry out feature selection”.

The idea of taking into account the topology structure of pathways seems to be very interesting and promising. However, I have doubts about the correctness of considerations given in the manuscript. Two arguments for such doubts are given below.

1. Pathway is a multidimensional structure. Dependences between genes in pathway are very essential. However, the authors use quantile normalization as a pre-processing procedure. It is known [41, 42] that quantile normalization destroys correlation structure between genes. Therefore, its application looks to be strange, and, at least, needs to be explained in more details.

Author’s response: *Thank you for this insightful comment. As demonstrated in* [41]*, quantile normalization may destroy both the spurious correlation structure and the true correlation structure between genes. This should have small impact on the weighted-SAMGSR algorithm given the weights in the weighted-SAMGSR algorithm were constructed on the basis of the PPI information, which was retrieved from major canonical databases, instead of the de novo networks constructed using the expression values.*

Furthermore, we have analyzed a non-small cell lung cancer RNA-Seq dataset using the expression profiles obtained by the Voom function (without quantile normalization). The conclusion that the weighted-SAMGSR algorithm is superior to the SAMGSR algorithm holds true in this application.

2. I do not see how pathway multidimensionality is used in the manuscript. The attempts to use it were proposed in [43]. I thing, the distance between pathways and genes in pathway is neither “statistical metric” nor Euclidean distance. Unfortunately, the choice of distance is not explained in the manuscript. Author just mentioned statistical metric without explanation of its suitability.

Basing on these arguments, I propose to the authors to add detailed explanations of the points mentioned above.

Author’s response: *The metrics in* [43] *are good statistics to evaluate the performance of a gene set analysis method. Nevertheless, this study focuses on the pathway-based feature/gene selection algorithms, which has essentially a different definition. While a gene set analysis method selects relevant pathways, a pathway-based feature selection algorithm identifies relevant genes but using pathway knowledge as a priori to guide the selection process.*

The statistical metrics we used in this study are suitable for evaluating the performance of a feature/gene selection algorithm. To clarify this, we have added their definitions in the Methods section and emphasized on their suitability to evaluate and compare the predictive performance of different feature/gene selection methods.

### Reviewer’s report 3: Dr. I. King Jordan, Georgia Institute of Technology, USA

#### Review summary

This manuscript reports a pathway-based feature selection method that incorporates prior biological information into the selection of a set of genes of interest based on the results of large-scale differential expression analysis between phenotypic conditions using microarrays. The method reported here is an extension of the previously developed significance analysis of microarray-gene set reduction algorithm (SAMGSR) from a different research group. The authors’ extended method entails a weighting step that takes pathway network topology into consideration and results in a reduction of the pathways selected for further analysis to their core constitutive genes, thereby yielding a more focused, and presumably more biologically relevant, subset of genes for subsequent analyses. It does this by incorporating a weight feature that is based on network connectivity (from protein-protein interactions in this case); genes with high connectivity are weighted more heavily in an effort to detect driver genes that may have more systemic influences on gene expression. This extension, while somewhat trivial, does seem to make intuitive sense, and the authors’ benchmarking analyses support its use.

#### Reviewer recommendations to authors

Methods of this kind are certainly of interest, in principle, to the biological research community and have the potential to better direct follow on experiments and eliminate wasted effort. My main concern is that given the fact that the manuscript is reporting an extension of an existing method, the bar is high with respect to both providing for and demonstrating the utility of the extended method. I feel that the authors should do more to 1) broaden the scope of their method, 2) demonstrate its utility, and 3) make it available to the research community.

1. The adjacency matrix used by the method is binary and allows genes to be connected (1) or not (0). It would be desirable to allow for edge weights to allow for the incorporation of confidence levels with respect to gene (protein) interactions.

Author’s response: *Thanks for the suggestion. Given the PPI information we downloaded from HPDB has only binary values (indicating if gene pairs are connected or not), we now also consider the STRING database to get the edge weights with confidence levels and apply the weighted-SAMGSR algorithm to demonstrate its utility. Please see Table*8*for the results of these analyses.*

2. The network connections used correspond to protein-protein interactions. However, there are many different kinds of interactions that contain biologically relevant information and are widely available. For example, the STRING database has different classes of protein-protein interactions as well as predicted interactions based on gene neighborhood, protein homology and text mining. The authors should explore the utility of different sources of gene (protein) interactions.

Author’s response: *We have added the analysis using the PPI information provided by the STRING database (using both confidence levels and binary values).*

3. The benchmarking analysis is limited to two microarray data sets (from the exact same array platform). RNA-seq is of course widely used for differential expression studies of the kind analyzed here, and the authors should also test one or two RNA-seq data sets.

Author’s response: *We have added more analyses using an RNA-seq non-small cell lung cancer dataset, downloaded from The Cancer Genome Atlas (TCGA) project.*

4. I could not find any indication of whether, or how, this method has been made available for the research community. The utility of this method would be greatly enhanced if the code, along documentation for how to use it, were released on a software repository such as GitHub. [Note that this can be done with the appropriate license if the authors are concerned about commercial applications].

Author’s response: *We are working on an R Bioconductor package including the weighted-SAMGSR algorithm (the work proposed in this manuscript) and two extensions to SAMGSR for longitudinal data analysis (the manuscript had been uploaded in Arxiv). We intend to present this package in a software paper.*

Nevertheless, in order to make the immediate use of the proposed methods by other researchers possible, we have added an Additional file 1 that presents the R program for the weighted-SAMGSR algorithm.

## Round 2

### Reviewer’s report 1: Dr. Limsoon Wong, National University of Singapore, Singapore

#### Reviewer comments to Authors

1/There is some problems in the PDF file. In particular, in many formula, some symbols are missing (they show up as empty boxes). As these formula are critical to understanding the proposed method and evaluate its correctness, I am unable to proceed reviewing the ms very carefully.

Author’s response: *Sorry the PDF file displayed these equations improperly. We had used an older version of Office on a MacBook, which somehow made the equations out of place. We have reformatted the Word file and addressed this problem.*

Nevertheless, a quick scan of the ms shows that most of my earlier comments have not been addressed very well. E.g.: 2/The point of my mentioning existing methods (like PFSNET, ESSNET, GSEA, Irizarry et al. and so on) is that the proposed method has not been compared with a convincing set of competing methods. It does not matter what approach classes these methods belong to, they are methods for solving the same problem as the proposed method. Hence to demonstrate how well the proposed method is doing against current methods, it is important see comparison against a broader range of existing methods.

Author’s response: *Besides LASSO and penalized SVM, we now have analyzed those datasets using two additional pathway-based feature selection algorithms — generalized elastic net (gelnet, a penalty method)* [25] *and reweighted recursive feature elimination (RRFE, a weighting method)* [14]*, and have compared these methods to the proposed weighted SAMGSR algorithm.*

As there are numerous existing feature selection algorithms, an exhaustive comparison between those methods and the weighted SAMGSR algorithm is impractical. We note that the sbv Improver challenge allowed comparison between some of the best-performed methods and our proposed method for the specific application.

3/The point of my mentioning the poor stability and cross-dataset error rate is that a good method should choose features that are reproducible and useable in future data. Otherwise the chosen features cannot be used in practice and are likely not causal to the phenotypes studied. That is, it is a signal that the results are not sound. Soundness is a crucial factor for/Biology Direct/in considering manuscripts. Hence the authors should properly investigate the poor stability and cross-dataset error rate of the proposed method, understand what causes this, and properly acknowledge and discuss the issue. The authors should make a further major revision to address these and other points raised in my previous review.

Author’s response: *First, we agree with the reviewer that chosen features with poor stability cannot be used in practice. Consequently, we don’t make any recommendation on the resulting gene or gene set signatures that should be used in practice. Our primary objective here is to introduce the weighted SAMGSR algorithm. Using simulations and multiple real-world applications, we have demonstrated that weighted SAMGSR is superior to the original SAMGSR algorithm.*

Second, regarding poor stability and predictive errors (based we note that these metrics are all data/application dependent. There are various examples to illustrate this. For example, in one article mentioned by the reviewer [37], PFSNet has an AUC of 0.556 and a recall of 0.667 on a colorectal cancer data whereas an AUC of 0.666 and a recall of 0.937 on another colorectal cancer data. Regarding stability, the between-dataset gene-set level agreement and gene-level agreement for PFSNet on the colorectal datasets are only 21.59 and 12.41 %, respectively. For different applications, the fact that gene expression profiles present different amounts of information and noises should be well acknowledged.

### Reviewer’s report 2: Dr. Lev Klebanov, Charles University, Czech Republic

#### Reviewer comments to authors

I have no additional comments. My previous comments were taking into account. All questions were answered.

### Reviewer’s report 3: Dr. I. King Jordan, Georgia Institute of Technology, USA

#### Reviewer comments to authors

I am satisfied that my original comments have now been addressed and recommend that the revised article be accepted for publication.

## Additional file

Additional file 1: R codes for the weighted-SAMGSR algorithm. (DOCX 76 kb)

## Abbreviations

AC: Adenocarcinoma
AUPR: Area under the precision-recall curve
BCM: Belief confusion metric
CV: Cross validation
GBS: Generalized brier score
GEO: Gene expression omnibus
MS: Multiple sclerosis
MSigDB: Molecular signatures database
NSCLC: Non-small cell lung cancer
SAM: Significance analysis of microarray
SAMGSR: Significance analysis of microarray gene set reduction
SCC: Squamous cell carcinoma
TCGA: The Cancer Genome Atlas
