# Supplementary material for: Weighted-SAMGSR: combining significance analysis of microarray-gene set reduction algorithm with pathway topology-based weights to select relevant genes
Source: Biol Direct. 2016 Sep 29;11:50. doi: 10.1186/s13062-016-0152-3 (PMC5041498; doi:10.1186/s13062-016-0152-3)
Supplement: Additional file 1: — R codes for the weighted-SAMGSR algorithm. (DOCX 76 kb) [file 13062_2016_152_MOESM1_ESM.docx]

Step 1: the weighted SAMGS function:

Weighted_SAMGS <- function(GS,

DATA,

cl,

nbPermutations=1000, PPI,

silent=F, a=1

, lambda=seq(0, 0.9, 0.05), pi0.method = "smoother"

){

GS <- GS.format.dataframe.to.list(GS)

if(!silent) print("GS dataframe-to-list : done.")

genes <- unique(intersect(dimnames(DATA)[[1]],unlist(GS)))

genes <- genes[!is.na(genes)] #####only the row numbers with gene in at least 1 set

nb.Samples <- ncol(DATA)

nb.GeneSets <- length(GS) # nb of gene sets

GeneSets.sizes <- sapply(GS,length) # size of each gene set

C1.size <- table(cl)[1] # nb of samples in class 1

DATA <- DATA[genes,]

# finding constant s0 for SAM-like test

tmp <- sam.TlikeStat(DATA,cl=cl)

s0 <- tmp$s0

if(!silent) print("s0 estimation : done.")

GS <- lapply(GS,function(z) which(genes %in% z))

# stats obtained on 'true' data

samT.ok <-as.data.frame(tmp$TlikeStat)[genes,] # SAM T-like statistic for each gene

weight<-(1+apply(PPI[genes, genes], 1, sum))^a

# weight<-sapply(GS, function(z) (1+apply(PPI[z,z], 1, sum))^a))

if (a==0) sam.sumsquareT.ok <- sapply(GS,function(z) sum(samT.ok[z]^2))

if (a!=0) sam.sumsquareT.ok <- sapply(GS,function(z) sum(weight[z]*samT.ok[z]^2))

# stats obtained on 'permuted' data

permut.C1 <- matrix(NA,nbPermutations,C1.size)

sam.sumsquareT.permut <- matrix(NA,nbPermutations,nb.GeneSets)

diperm<-matrix(NA,(dim(DATA)[1]), (nbPermutations+1))

diperm[,1]<-tmp$TlikeStat

for(i in 1:nbPermutations) {

C1.permut <- permut.C1[i,] <- sample(nb.Samples,C1.size)

C2.permut <- (1:nb.Samples)[-C1.permut]

samT.permut <- sam.TlikeStat(DATA,C1=C1.permut,C2=C2.permut,s0=s0)$TlikeStat

if (a==0) sam.sumsquareT.permut[i,] <- sapply(GS,function(z) sum(samT.permut[z]^2))

if (a!=0) sam.sumsquareT.permut[i,] <- sapply(GS,function(z) sum(weight[z]*samT.permut[z]^2))

diperm[,(i+1)] <-samT.permut

}

GeneSets.pval <- apply(t(sam.sumsquareT.permut) >=sam.sumsquareT.ok ,1,sum)/nbPermutations

qobj <- NULL

try(qobj <- qvalue(GeneSets.pval, lambda=lambda))

GeneSets.qval <- rep(NA,nb.GeneSets)

PI0 <- NA

if(!is.null(attr(qobj,"class"))){

GeneSets.qval <- qobj$qvalues

PI0 <- qobj$pi0

}

res <- as.data.frame(cbind("GS size" = GeneSets.sizes,

"statistic value" =sam.sumsquareT.ok,

"GS p-value (0 <=> < 1/nb permutations)" = GeneSets.pval,

"GS q-value"= GeneSets.qval))

res <- cbind(res,"GS name"= names(GS))[c(4:5,1:3)]

L <- list("GS stats"=res,

"diperm"=diperm,

"genes stats"= as.data.frame(cbind(tmp[[4]],tmp[[5]])))

L

}

Step 2: The reduction step.

out1<-out[[1]]

rt<-dim(out1[out1[,5]<0.05,])[1]

c=0.05 #the cutoff for ck

simresults=list()

redset=list()

redset.name<-list()

redsetsizec=matrix(NA,rt,length(c))

diperm<-out[[2]]

rownames(diperm)<-rownames(DATA)

GS.name<-out1[,1]

GS.name<-GS.name[order(pval)]

GS.select[[j]]<-GS.name[1:rt]

noperm<-201

for (op in 1:rt){

gsi=order(pval)[op]

digsi=diperm[restrictmapgenes[,gsi]==1,] #select dimatrix for gene set of interest

rownames(digsi)<-rownames(DATA)[which(restrictmapgenes[,gsi]==1)]

dim(digsi)

digsisq=digsi**2

order(-digsisq[,1]) #order the genes according to their significance.

odigsisq=digsisq[order(-digsisq[,1]),]

redset.name[[op]]<-rownames(odigsisq)

#Reduce gene set

#calculate gene set red p-val

ns=nrow(odigsisq) #set size

print(paste("ns",ns))

gsredpval=rep(0,(ns-1)) #pbar

gsredpvalR=rep(0,(ns-1))

samgs=rep(NA,ns)

for(g in 1:(ns-1)){

for (i in 2:noperm){

if (sum(odigsisq[(g+1):ns,i])>=sum(odigsisq[(g+1):ns,1])) gsredpval[g]=gsredpval[g]+1 #pbar

if (sum(odigsisq[1:g,i])>=sum(odigsisq[1:g,1])) gsredpvalR[g]=gsredpvalR[g]+1

samgs[g]=sum(odigsisq[(g+1):dim(odigsisq)[1],1])

}

}

gsredpval=gsredpval/(noperm-1) #pbar-value #CK

gsredpvalR=gsredpvalR/(noperm-1) #p-value

#redsetsize based on pbar cutoff

if (.05>=max(gsredpval)) redsetsizec[op,cit]=ns else redsetsizec[op,cit]=1+sum(c[cit]>=gsredpval)

print(redsetsizec[op,cit])

gspval=cbind(gsredpvalR,gsredpval)

simresults[[op]]=gspval

redset[[op]]=rownames(Gender)[restrictmapgenes[,gsi]==1][order(-digsisq[,1])][1:(redsetsizec[op,cit])]

redset.name[[op]]<-redset.name[[op]][1:length(redset[[op]])]

}# end of op iterations

unique(unlist(redset.name))
